# UMP Kinase Regulates Chloroplast Development and Cold Response in Rice

**DOI:** 10.3390/ijms20092107

**Published:** 2019-04-29

**Authors:** Qing Dong, Ying-Xin Zhang, Quan Zhou, Qun-En Liu, Dai-Bo Chen, Hong Wang, Shi-Hua Cheng, Li-Yong Cao, Xi-Hong Shen

**Affiliations:** State Key Laboratory of Rice Biology and Key Laboratory for Zhejiang Super Rice Research, China National Rice Research Institute, Hangzhou 310006, China; dongqing66job@sina.com (Q.D.); zhangyingxin@caas.cn (Y.-X.Z.); stresszhou@163.com (Q.Z.); liuqunen202@163.com (Q.-E.L.); cdb840925@163.com (D.-B.C.); wjiyinh@126.com (H.W.); chengshihua@caas.cn (S.-H.C.)

**Keywords:** UMP kinase, chloroplast development, abiotic stress response, rice

## Abstract

Pyrimidine nucleotides are important metabolites that are building blocks of nucleic acids, which participate in various aspects of plant development. Only a few genes involved in pyrimidine metabolism have been identified in rice and the majority of their functions remain unclear. In this study, we used a map-based cloning strategy to isolate a *UMPK* gene in rice, encoding the UMP kinase that phosphorylates UMP to form UDP, from a recessive mutant with pale-green leaves. In the mutant, UDP content always decreased, while UTP content fluctuated with the development of leaves. Mutation of *UMPK* reduced chlorophyll contents and decreased photosynthetic capacity. In the mutant, transcription of plastid-encoded RNA polymerase-dependent genes, including *psaA*, *psbB*, *psbC* and *petB*, was significantly reduced, whereas transcription of nuclear-encoded RNA polymerase-dependent genes, including *rpoA*, *rpoB*, *rpoC1*, and *rpl23*, was elevated. The expression of *UMPK* was significantly induced by various stresses, including cold, heat, and drought. Increased sensitivity to cold stress was observed in the mutant, based on the survival rate and malondialdehyde content. High accumulation of hydrogen peroxide was found in the mutant, which was enhanced by cold treatment. Our results indicate that the UMP kinase gene plays important roles in regulating chloroplast development and stress response in rice.

## 1. Introduction

Pyrimidine nucleotides are essential for plants. They are not only the building blocks for nucleic acid synthesis, but also provide precursors for a wide range of cellular components, such as sugar, polysaccharides, glycoproteins, and phospholipids [1,2]. Pyrimidine metabolism in plants can be broadly divided into four pathways: *de novo* synthesis that produces UMP; nucleotide inter-conversions that modify UMP to form other pyrimidines; salvaging reactions that recycle nucleosides and free bases; and catabolism that degrades pyrimidines into simple cellular metabolites [2]. Levels of pyrimidine nucleotides depend on the interaction and coordination of enzymes involved in these pathways.

More than 20 enzymes are involved in pyrimidine metabolism in plants [1,2], whereas only a few genes encoding these enzymes have been identified by molecular genetic analysis in rice. *OsDHODH1* encodes a cytosolic dihydroorotate dehydrogenase in the *de novo* pathway, which catalyzes the conversion of dihydroorotate to form orotate. Overexpression of *OsDHODH1* enhanced plant tolerance to salt and drought stresses [3]. *OsNDPK2* encodes a nucleoside diphosphate kinase in the nucleotide inter-conversion pathway, which catalyzes the phosphorylation of NDP to form NTP. Mutation of *OsNDPK2* impaired chloroplast development and increased sensitivity to salinity stress [4,5]. *ST2*/*ALR* encodes a dCMP deaminase in the nucleotide inter-conversion pathway, which catalyzes dCMP to dUMP. Mutation of *ST2*/*ALR* impaired chloroplast development and retarded growth [6,7].

Genes encoding other enzymes involved in pyrimidine metabolism have been isolated in other plant species, including carbamoylphosphate synthase, aspartate transcarbamoylase, UMP synthase, adenylate kinase, CTP synthase, nucleoside triphosphate phosphatase, apyrase, uridine kinase, uridine ribohydrolase, cytidine deaminase, and dihydropyrimidine dehydrogenase [8,9,10,11,12,13,14,15,16,17,18,19]. These genes were found to regulate seed germination, chloroplast biogenesis, development of root and seed, response to biotic and abiotic stresses, and so on. These studies illustrated that the genes involved in pyrimidine metabolism play important roles in various aspects of plant development.

The UMP kinase is a key enzyme in the nucleotide inter-conversion pathway that phosphorylates UMP to form UDP [1,2]. Recent studies revealed that UMP kinase is involved in chloroplast development in plants. Mutation of the UMP kinase gene impaired development of photosystem I and affected the accumulation of *psaA/B* transcripts in *Arabidopsis* [20]. Two mutants of the UMP kinase gene were isolated in rice, *ygl8* and *yl2*, in which reduced chlorophyll contents, decreased photochemical efficiency, and abnormal chloroplast ultrastructure were observed [21,22]. However, transcription of the genes needed for chloroplast development varied between the two mutants. In the *ygl8* mutant, the expression of most of chlorophyll biosynthesis genes and plastid genes was significantly reduced, compared with wild-type. In the *yl2* mutant, the transcription levels of genes required for chloroplast development were significantly elevated or unchanged. Therefore, the exact mechanism associated with the effects of mutation of the UMP kinase gene on chloroplast development remains to be explored. In addition, several studies have implicated some of the genes involved in the pyrimidine metabolism pathway with the response of plants to abiotic stresses [3,4,12,13,14]. However, little is known about the role of the UMP kinase gene.

In the present study, we identified a recessive rice mutant, *umpk*, with pale-green leaves throughout the entire growth period. We isolated the *UMPK* gene with a map-based cloning strategy and demonstrated that it encodes the UMP kinase, which affected the synthesis of UDP and UTP. Mutation of *UMPK* disrupted chloroplast development, likely resulting from impairment of the transcription activity of the plastid-encoded RNA polymerase. In addition, mutation of *UMPK* increased sensitivity to cold stress, likely due to the over-accumulation of reactive oxygen species. Our results indicate that the UMP kinase gene plays important roles in chloroplast development and abiotic stress response in rice.

## 2. Results

### 2.1. Phenotype Characterization of the umpk Mutant

**Figure 1:**
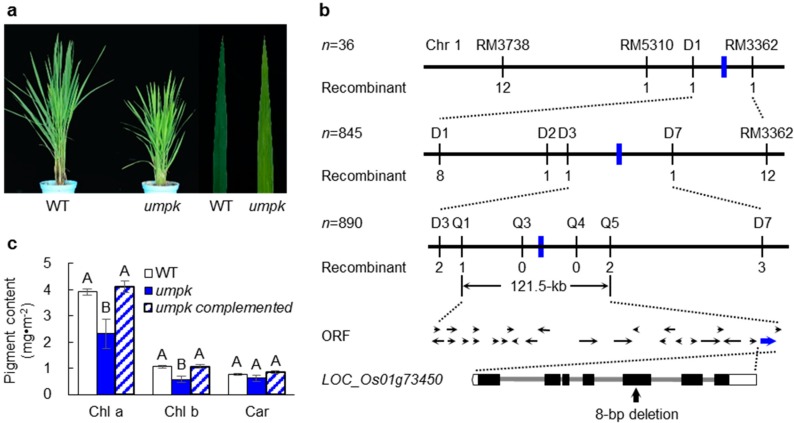
Map-based cloning of *UMPK*. (**a**) Phenotypes of the two-month-old wild type (WT) and mutant. (**b**) Map-based cloning of *UMPK* locus. (**c**) Chlorophyll contents of the WT, the mutant, and transgenic lines. Data represent means ± sd (*n* = 5). Bars with different letters are significantly different at *p* < 0.01 based on Duncan’s multiple range tests.

The *umpk* mutant was identified from a mutagenized population of the *indicia* rice variety Zhonghui 8015 (WT) treated with ethyl methanesulfonate (EMS). Leaves of *umpk* exhibited a pale-green phenotype throughout the entire growth period (Figure 1a). Photosynthetic pigments were measured in six-week-old WT and mutant plants. Contents of chlorophyll a and chlorophyll b clearly decreased in the mutant compared with WT, while no significant difference was detected for carotenoid between the two genotypes (Figure S1a). The light-induced P700 absorbance changes at 820 nm (*∆I/Io*), the actual quantum efficiency (*ΦPSII*), and photosynthesis rate were also measured. All of them were significantly decreased in the mutant (Figure S1b). The ultrastructure of chloroplasts was further investigated (Figure S1c). In the WT, chloroplasts showed normal morphology. However, the chloroplasts in the mutant showed an abnormal morphology, with deformed thylakoid membranes and poorly stacked grana. These findings indicated that chloroplast development was impaired in the *umpk* mutant.

### 2.2. Map-based Cloning of the UMPK Gene

The *umpk* mutant was crossed with cultivar 02428. The resultant F_2_ population segregated in a 3 normal: 1 mutant ratio (green: pale green = 774: 272, *χ*^2^ = 0.51 < *χ*^2^_0.05_ = 3.84, *p* = 0.4534), suggesting that the *umpk* mutant phenotype was due to a single recessive nuclear gene. Bulked segregant analysis revealed that four markers on the long arm of chromosome 1 co-segregated with the phenotype of *umpk* (Figure 1b). Plants with the recessive phenotype, including 845 F_2_ plants and 890 F_3_ plants, were further genotyped. The *UMPK* gene was finally narrowed down to a 121.5-kb region flanked by markers Q1 and Q5.

This region contains 18 annotated genes (Figure 1b), according to the Rice Genome Annotation Project Database (http://rice.plantbiology.msu.edu). The coding regions of these genes were amplified from the WT and the mutant, and then sequenced. An 8-bp deletion was found in the fifth exon of *LOC_Os01g73450* in the mutant, leading to a frame shift. *LOC_Os01g73450*, also known as *YGL8*/*YL2*, has been previously reported to encode a UMP kinase and to be involved in regulating rice chloroplasts [21,22]. A genetic complementation experiment was performed by introducing a genomic fragment harboring the entire *UMPK* gene of the WT into the mutant. The phenotype and Chl contents of the mutant were restored to normal in the positive transgenic plants (Figure 1c, Figure S1d). Thus, *UMPK* was the same gene as *YGL8*/*YL2*.

### 2.3. Nucleotide Synthesis Was Affected in the umpk Mutant

**Figure 2:**
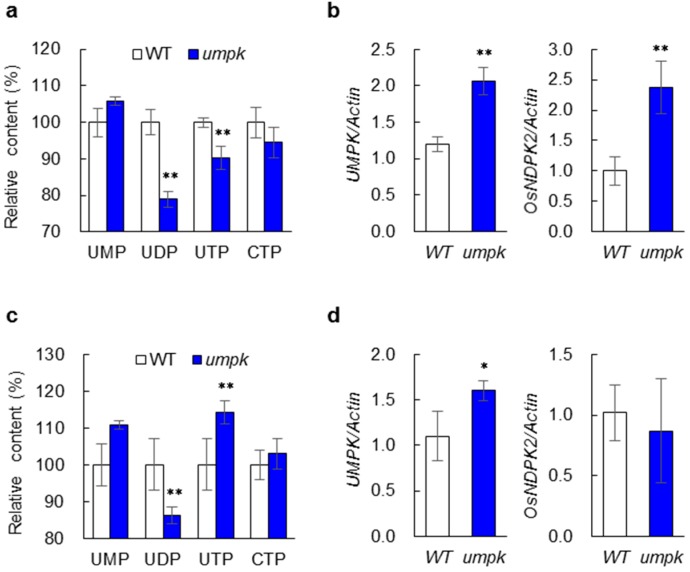
Pyrimidine metabolism analysis in the WT and the *umpk* mutant. (**a**) Pyrimidine nucleotides levels in the youngest fully expanded leaves. (**b**) Expression of two genes involved in pyrimidine metabolism in the youngest fully expanded leaves. *Actin1* was used as the internal control. (**c**) Pyrimidine nucleotides levels in old leaves. (**d**) Expression of two genes involved in pyrimidine metabolism in old leaves. *Actin1* was used as the internal control. Data represent means ± sd (*n* = 3). Significant differences are according to the Student’s *t*-test at * *p* < 0.05 and ** *p* < 0.01.

The UMP kinase has been demonstrated to catalyze the phosphorylation of UMP to form UDP. UDP is converted to UTP and then to CTP [1,2]. Therefore, contents of these pyrimidine nucleotides were measured in young leaves of the six-week-old WT and mutant plants. Significant differences were detected for UDP and UTP, but not for UMP and CTP (Figure 2a). Compared with WT, UDP and UTP in the mutant decreased by 21.0% and 9.7%, respectively. These decreases suggest that the mutation of *UMPK* affected the pyrimidine synthesis and the influence was greater on the direct product UDP than on the indirect product UTP. Expression analyses were performed for *UMPK* and another gene, *OsNDPK2*, which encodes the nucleoside diphosphate kinase that phosphorylates UDP to form UTP [4,5] (Figure 2b). The two genes exhibited higher expression levels in the mutant, more than twice those in the WT.

We further examined pyrimidine contents in older leaves. The UDP content was still lower in the mutant than in the WT, but the extent of reduction (13.7%) was smaller than that observed in young leaves. In contrast to the results observed in the young leaves, the UTP content in the mutant increased compared with WT (14.3%) (Figure 2c). Expression levels of the two pyrimidine synthesis genes, *UMPK* and *OsNDPK2*, were also investigated. A significant difference was only detected for *UMPK*, which increased by 45.5% in the mutant compared with WT (Figure 2d). These results suggested that the *umpk* mutant might up-regulate the expression of genes involved in pyrimidine synthesis to remedy the influence caused by the mutation of *UMPK*.

### 2.4. Plastid Transcription Was Altered in the umpk Mutant

**Figure 3:**
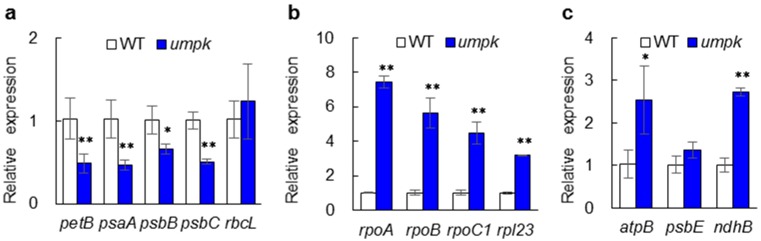
Expression of plastid genes in the WT and the *umpk* mutant. (**a**) Plastid-encoded RNA polymerase (PEP)-dependent genes. (**b**) Nucleus-encoded RNA polymerase (NEP)-dependent genes. (**c**) Both PEP- and NEP-dependent genes. *Actin1* was used as an internal control. Data represent means ± sd (*n* = 3). Significant differences are according to the Student’s *t*-test at * *p* < 0.05 and ** *p* < 0.01.

To investigate if plastid transcription was impaired by the mutation of *UMPK*, the expression of plastid genes was examined in young leaves of six-week-old WT and mutant plants. The genes *psaA*, *psbB*, *psbC, petB*, and *rbcL* were selected as plastid-encoded RNA polymerase (PEP)-dependent genes (Figure 3a); *rpoA*, *rpoB*, *rpoC1*, and *rpl23* were selected as nucleus-encoded RNA polymerase (NEP)-dependent genes (Figure 3b); *atpB*, *ndhB*, and *psbE* were chosen as both PEP- and NEP-dependent genes (Figure 3c) [23,24,25,26]. The expression of all assayed PEP-dependent genes was significantly lower in the mutant, except for *rbcL*, which showed no significant difference between the WT and the mutant. Compared with WT, the expression levels of *psaA*, *psbB*, *psbC*, and *petB* were reduced by 54.2%, 34.3%, 49.9%, and 52.3% in the mutant, respectively. On the contrary, NEP-dependent genes were significantly up-regulated. The expression levels of *rpoA*, *rpoB*, *rpoC1*, and *rpl23* in the mutant were 6.4, 5.5, 4.3, and 2.2 times greater than the WT, respectively. The genes, *atpB* and *ndhB*, which are both PEP- and NEP-dependent, were also up-regulated in the mutant. No significant difference between the WT and the mutant was observed for *psbE*. These results demonstrate a typical plastid gene expression pattern caused by impaired PEP transcription, suggesting that mutation of the *UMPK* gene impaired transcription by the plastid-encoded RNA polymerase.

The expression of the plastid genes was further examined in the leaves of the one-week-old WT, the complementation plants, and the mutant plants. Compared with complementation plants, in the mutant, the four PEP-dependent genes were significantly down-regulated, while all the NEP-dependent genes were significantly up-regulated (Figure S2). These results confirmed that the transcription of plastid-encoded genes was impaired by mutation of *UMPK*.

### 2.5. Sensitivity to Abiotic Stress Increased in the umpk Mutant

**Figure 4:**
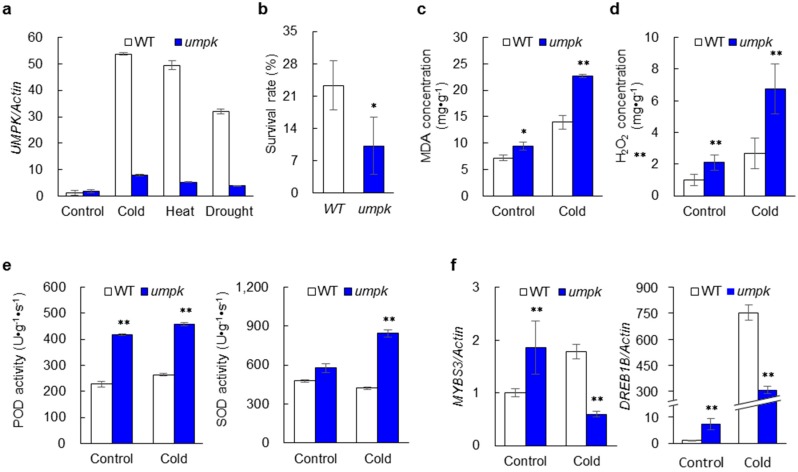
Response of *UMPK* to abiotic stresses. (**a**) Expression levels of *UMPK* under various stress treatments in the WT and the mutant (*n* = 3). *Actin1* was used as the internal control. (**b**) Survival rates of the WT and the mutant after cold treatment (3 replicates, 30 plants in each replicate). (**c**) Concentrations of malondialdehyde (MDA) in the WT and the mutant under normal and cold conditions (*n* = 5). (**d**) Concentrations of hydrogen peroxide (H_2_O_2_) in the WT and the mutant under normal and cold conditions (*n* = 5). (**e**) Activities of peroxidase (POD) and superoxide dismutase (SOD) in the WT and the mutant under normal and cold conditions (*n* = 5). (**f**) Expression levels of two cold-inducible genes in the WT and the mutant under normal and cold conditions (*n* = 3). *Actin1* was used as the internal control. Data represent means ± sd. Significant differences are according to the Student’s *t*-test at * *p* < 0.05 and ** *p* < 0.01.

The four-week-old WT and mutant were treated with various stresses, including cold (6 °C, 2 days), heat (45 °C, 1 day), and drought (20% PEG-6000, 4 days) conditions. The transcript level of *UMPK* was significantly elevated by all the three stresses, and the effects were much larger in the WT than the mutant (Figure 4a). Compared with normal conditions (28 °C), the expression of *UMPK* under cold, heat, and drought conditions increased by 53.8, 49.5, and 32.0 times in the WT, respectively, while it only increased by 4.4, 2.9, and 2.1 times in the mutant, respectively. Similar results were also observed in the one-week-old WT, the complementation plants, and the mutant plants. Compared with normal conditions, the expression of *UMPK* under the three stresses increased by 22.0−94.2 times in the WT and the complementation plants, but it only increased by 1.3−5.8 times in the mutant (Figure S3a). These results suggest that the expression of *UMPK* was induced by various abiotic stresses, and this transcriptional response was impaired in the mutant.

The four-week-old WT and mutant plants subjected to cold treatment were transferred to normal conditions and survival rates were investigated after two weeks (Figure 4b). Compared with WT, survival rate decreased by 56.2% in the mutant, indicating that the mutation of *UMPK* increased cold sensitivity. Malondialdehyde (MDA) and hydrogen peroxide (H_2_O_2_) were measured under normal and cold conditions (Figure 4c,d). Significant differences were detected between the WT and the mutant, which was larger under cold conditions than normal conditions. Compared with the WT, the level of MDA in the mutant was higher by 31.4% under control conditions, while the level was higher by 63.1% under cold conditions. The level of H_2_O_2_ in the mutant was higher by 110.5% compared with WT under control conditions, but it was higher by 152.1% under cold conditions.

Activities of scavengers of reactive oxygen species (ROS), including superoxide dismutase (SOD) and peroxidase (POD), were determined (Figure 4e). For POD, significant differences were detected between the WT and the mutant under the respective cold-stress and control conditions. Compared with the WT, the activities of POD in the mutant were higher by 83.4% and 74.2% under normal and cold conditions, respectively. For SOD, a significant difference between the two genotypes was only detected under cold conditions. The activity was higher by 100.1% in the mutant compared to the WT. The expression levels of the two cold-stress response genes, *DREB1B* and *MYBS3* [27,28,29], were investigated (Figure 4f). Under control conditions, the expression levels of the two genes were significantly higher in the mutant than the WT. Conversely, they were significantly lower in the mutant than in the WT under cold conditions.

## 3. Discussion

In the present study, we identified a recessive rice mutant, *umpk*, which displayed pale-green leaves throughout its entire growth cycle. Map-based cloning showed that *UMPK* encoded a UMP kinase involved in nucleotide metabolism, corresponding to *umpk*, and was the same gene as the *YGL8*/*YL2* genes previously reported by Zhu et al. [21] and Chen et al. [22]. These two studies revealed that mutation of the UMP kinase gene reduced Chl contents, led to abnormal ultrastructure of chloroplasts, and impaired photosynthetic capacity. These effects were also observed in our study, which demonstrates that the UMP kinase is essential for chloroplast development.

The mutations of *YGL8* and *YL2* exhibited some distinct molecular features. The transcription levels of most nucleus- and plastid-encoded photosynthetic genes were significantly reduced in the *ygl8* mutant compared with WT; however, the expression levels of those genes in the *yl2* mutant were elevated or unchanged. Expression of plastid genes depends on the activity of two types of RNA polymerases: PEP transcribes genes that are largely involved in photosynthesis, whereas NEP mainly transcribes housekeeping genes [30,31]. In the present study, the expression levels of plastid genes were investigated. The results showed that transcript levels of PEP-dependent genes and NEP-dependent genes in the *umpk* mutant were significantly reduced and elevated, respectively, compared with WT and the complementation lines. This is a typical plastid gene expression pattern resulting from impaired PEP transcription, which frequently results in abnormal chloroplast development [23,26,32,33,34,35,36,37]. The influence of impaired PEP activity on chloroplast development varied during the development of plants: it was more severe at the early stage and gradually diminished with plant development. Phenotypic changes during plant development were also observed in the *yl2* mutant [22]. Therefore, the differences in molecular features between these three allelic mutations might be the result of sampling at different development stages. Our results revealed that *UMPK* plays an important role in maintaining PEP transcription activity and the inefficiency of chloroplast development in the *umpk* mutant might be associated with the impairment of PEP activity.

Three other genes involved in nucleotide metabolism were also found to regulate PEP activity. They included *OsNDPK2* [5]; *ATase2* encoding glutamine phosphoribosyl pyrophosphate amidotransferase that catalyzes the first step of purine biosynthesis [26]; and *GARS* encoding a glycinamide ribonucleotide synthetase that catalyzes the second step of purine biosynthesis [33]. In mutants of these genes, the expression of PEP-dependent genes was consistently down-regulated. Since pyrimidine and purine nucleotides are substrates for nucleic acid synthesis, it was speculated that the down-regulation of PEP-dependent genes is caused by a shortage of nucleotides in these mutants. However, up-regulation of NEP-dependent genes was also detected in the *umpk* mutant in the present study and the mutant of *ATase2* [26]. Therefore, the relationship between the genes involved in nucleotide metabolism and plastid transcription may be more complicated.

More recently, the UMP kinase in *Arabidopsis* was found to be an RNA binding protein that has an impact on the accumulation of its plastid RNA targets. One of its targets, *trnG*-UCC, is essential for translation. Mutation of the UMP kinase gene led to a decrease of *trnG*-UCC, which in turn impaired the translation of chloroplast genes [38]. In the present study, the decrease of PEP activity in the *umpk* mutant might be caused by insufficient translation, because the core subunits of PEP are encoded by the chloroplast genes, including *rpoA*, *rpoB*, *rpoC1*, and *rpoC2*. For the increase of NEP activity in the *umpk* mutant, which is typically observed in PEP mutants [23,26,32,33,34,35,36,37], there are at least two alternative explanations. First, the core subunits of NEP are encoded by nucleus genes; thus, NEP activity could not be affected by insufficient translation in chloroplasts. Conversely, the *umpk* mutant might up-regulate NEP activity to compensate for the decrease of PEP activity, because the core subunits of PEP are mainly transcribed by NEP. Second, RNA polymerase usage is found to switch from NEP to PEP during chloroplast development in higher plants. High activity of NEP is observed during the early stage. With the development of chloroplasts, the activity of PEP subsequently increases. One product of PEP, tRNA^Glu^, could repress NEP activity, leading to the switch from NEP to PEP [39]. The impairment of PEP activity in the *umpk* mutant might cause tRNA^Glu^ shortage, leading to de-repression of NEP activity.

Pyrimidine nucleotides are one of the most fundamental cellular components and have essential functions in all living organism. The UMP kinase is a crucial enzyme of pyrimidine metabolism. The kinase activity of this enzyme in rice has also been confirmed in vitro [22]. In the present study, the substrate and products of the UMP kinase were measured in the WT and the *umpk* mutant. The results showed that the UDP level was clearly reduced in the *umpk* mutant compared with WT, suggesting that *UMPK* encodes the enzyme that phosphorylates UMP to yield UDP. In the nucleotide inter-conversion pathway, UDP is further converted to UTP, which is catalyzed by nucleoside diphosphate kinases [1,2]. Our results showed that expression levels of *UMPK* and a nucleoside diphosphate kinase gene *OsNDPK2* were higher in young leaves of the *umpk* mutant. Consistently, the magnitude of reduction of UDP was decreased, and UTP was even highly expressed in old leaves of the mutant. These findings suggest that pyrimidine metabolism is regulated by an intricate network in rice, which equilibrates different pools of nucleotides.

Several genes associated with pyrimidine metabolism are involved in the response of plants to abiotic stresses [3,4,12,13,14], but little is known about the role of the UMP kinase gene. In the present study, *UMPK* expression was induced by cold, heat and drought treatment, which was much more obvious in the WT. Furthermore, the mutation of *UMPK* decreased the survival rate and enhanced MDA accumulation under cold conditions. In addition, the expression of the two positive regulators of cold tolerance, *DREB1B* and *MYBS3*, was significantly lower in the *umpk* mutant than the WT under cold conditions. Moreover, strong induction of *UMPK* and the two cold-inducible genes were also observed in the complemented transgenic lines (Figure S2), though we did not investigate the survival rate and MDA accumulation in the transgenic lines. These results clearly indicate that *UMPK* is a positive factor in response to cold stress. *DREB1B* has been shown to respond quickly and transiently whereas *MYBS3* responds slowly to cold stress in rice [29]. Low transcript levels were observed for both genes in the mutant under cold conditions, implying that *UMPK* is most likely crucial for responses to both shock and persistent cold stress.

ROS, such as singlet oxygen and H_2_O_2_, are by-products of various metabolic pathways and are also induced by stress conditions [40]. In general, lower doses of ROS act as molecular signals in response to abiotic stress, while higher concentrations of ROS act as toxic metabolic products in response to stresses with negative effects on plant development. Scavengers of ROS, such as SOD and POD, play important roles in maintaining appropriate ROS levels. In our study, H_2_O_2_ was highly accumulated in the *umpk* mutant under normal conditions. This indicates that the UMP kinase is critical for maintaining appropriate ROS metabolism in rice, which is similar to four other enzymes involved in nucleotide metabolism, including nucleoside diphosphate kinase 1, nucleoside diphosphate kinase 2, glutamine phosphoribosyl pyrophosphate amidotransferase 2, and allantoinase [4,41,42,43]. In addition to the high accumulation of ROS, high activities of SOD and POD were also observed in the mutant under normal conditions. The activities of SOD and POD increased but the mutant still had higher ROS accumulation, implying that the mutant cannot produce sufficient ROS scavengers to reduce the ROS even under normal conditions. When the mutant was exposed to cold stress, a further increase in ROS consequently led to increased sensitivity of the *umpk* mutant to cold stress. Therefore, the over-accumulation of ROS caused by low levels of UMP kinases might be a reason why the sensitivity to cold stress increased in the *umpk* mutant.

Chloroplasts are the main site of ROS production in plants and act as sensors of environmental stresses [40]. An increase of ROS levels has been frequently observed in mutants with impaired chloroplast functions [4,41,42,43]. Accordingly, the over-accumulation of ROS and increase of cold sensitivity in the *umpk* mutant might be an indirect effect of abnormal chloroplast ultrastructure and photosynthetic activity. However, we cannot rule out the possibility that *UMPK* regulates ROS production through another pathway. It should be noted that the expression of *NDPK2* increased in the *umpk* mutant. *Arabidopsis AtNDPK2* has been implicated in ROS signaling through its interaction with ROS regulators, including *AtMPK3*, *AtMPK6*, and *SOS2* [44,45]. Therefore, *UMPK* could be involved in the regulation of ROS levels through regulation of pyrimidine metabolism. Further biochemical and molecular studies are required to elucidate the exact roles of *UMPK* in the regulation of ROS accumulation and cold stress response.

## 4. Materials and Methods

### 4.1. Plant Material and Growth Conditions

The *umpk* mutant was obtained from a mutant population of *indica* rice cultivar Zhonghui 8015 after EMS treatment. For the EMS treatment, seeds soaked for 12 h in water were transferred into a 1.0% EMS solution for 12 h, followed by a 12 h wash using flowing water to accelerate germination. Rice materials were tested in the experimental stations of the China National Rice Research Institute located at Hangzhou in Zhejiang Province. For experiments (except for abiotic stress treatments), rice lines were grown in a paddy field following standard management practices. For abiotic stress treatments, rice lines were grown in hydroponic culture with a nutrient solution in a chamber with 12-h light/12-h dark cycles. The composition of the nutrient solution was the same as described in Yoshida et al. [46]. Light was provided by fluorescent lamps with 220 μmol m^−2^ s^−1^ of light intensity. The plants were grown at 28 °C for about 4 weeks (WT and mutant plants) or about 5 days (WT, complementation plants, and mutant plants), and then treated with abiotic stresses, including cold (seedlings were transferred to 6 °C for 2 days), heat (seedlings were transferred to 45 °C for 1 day), and drought (20% PEG-6000 was added to the hydroponic culture medium for 4 days).

### 4.2. Measurement of Pigment Content, Photochemical Efficiency of Photosystem, and Photosynthetic Rate, and Transmission Electron Microscopy Assay

The youngest fully expanded leaves from the six-week-old WT and mutant were used. The contents of pigments, including Chl a, Chl b, and carotenoid, were determined using a spectrophotometer (DU800, Beckman, Brea, California, USA), according to the method of Lichtenthaler [47]. The photochemical capacity of PSI was determined as *∆I/Io*, according to the method of Zhang et al. [48]. The photochemical capacity of PSII was determined as *ΦPSII* using a PAM-2500 portable chlorophyll fluorometer (Heinz Walz, Forchheim, Germany), according to the method of Zhao et al. [49]. The photosynthetic rate was determined at 9:00−11:00 on a sunny day using the portable photosynthesis measurement device LI-6400 (LICOR, Lincoln, Nebraska, USA), according to the method of Zhao et al. [49]. Transmission electron microscopy was performed according to the method of Li et al. [50].

### 4.3. Map-based Cloning of UMPK

The *umpk* mutant was crossed with rice cultivar 02428 to construct a segregating population. For genetic tests, 1046 plants were randomly selected from the F_2_ population containing more than 3000 individuals for phenotype analysis. For preliminary mapping, a bulk segregant analysis method [51] was initially used. The mutant and 02428 were assayed with 436 SSR markers and 91 Indel markers, and a total of 110 markers showing polymorphism between the two varieties were identified. Two phenotypic bulks, one consisting of 10 individuals with the WT phenotype and another consisting of 10 individuals with the pale green phenotype, were selected from the F_2_ population. The two bulks were assayed with the polymorphic markers to determine if the markers co-segregated with the phenotype of *umpk*. To validate these co-segregating markers, 36 F_2_ plants with the recessive pale green phenotype were genotyped. For fine-mapping, additional polymorphic InDel markers between the mutant and 02428 were developed to assay an additional 809 F_2_ plants; 890 F_3_ plants with the recessive pale green phenotype were assayed with the markers surrounding the preliminary linkage locus.

To analyze sequences of annotated genes located in the fine-mapping region, the coding regions of these genes were amplified from the WT and the mutant, and then sequenced. For complementation, a 6916 bp genomic fragment containing an entire open reading frame, 1519 bp upstream of the start codon, and 1616 bp downstream of the termination codon for *UMPK* was amplified from the WT. The PCR product was recombined into the binary vector pCAMBIA1300 using an In-Fusion Advantage Cloning kit (Takara, Japan). The resultant expression construct was introduced into the *umpk* mutant by *Agrobacterium*-mediated transformation. SSR markers were selected from the Gramene database (http://www.gramene.org). The InDel markers used for bulk segregant analysis were selected from the study of Wu et al. [52]. The InDel markers used for fine-mapping were developed according to differences between the genome sequence of *japonica* Nipponbare and *indica* 9311 (http://www.gramene.org). The primers used for sequence analysis and construction of complementation vector were developed according to the sequence of Nipponbare. The primers developed in this study are listed in Table S1.

### 4.4. RNA Extraction and Quantitative Real-Time PCR Analysis

To investigate the expression of pyrimidine synthesis genes, young leaves (the youngest fully expanded leaves) and old leaves (the fourth leaf from the top) were collected from the six-week-old WT and mutant. To investigate the expression levels of plastid genes, young leaves were collected from the six-week-old WT and mutant, or leaves were collected from the one-week-old WT, complementation lines, and the mutant. To examine the expression of *UMPK* or cold-inducible genes under normal and abiotic stresses conditions, young leaves were collected from the four-week-old WT and mutant, or leaves were collected from about one-week-old WT plants, complementation lines, and the mutant. Total RNA was extracted from these rice tissues using an RNeasy Plus Mini Kit (QIAGEN, German). First-strand cDNA was synthesized using a ReverTra AceR kit (Toyobo, Japan). Quantitative real-time PCR was performed on an Applied Biosystems 7500 using SYBR qPCR Mix kit (Toyobo, Japan). *Actin1* was used as the endogenous control. Data were analyzed according to the 2*^−ΔΔCt^* method [53]. Three independent biological replicates, each consisting of five plants for tissue samples, and three technical replicates per biological replicate were used. Primers are listed in Table S1.

### 4.5. Nucleotide Measurement

New leaves (the youngest fully expanded leaves) and old leaves (the fourth leaf from the top) were collected from the six-week-old WT and mutant. Samples were frozen immediately in liquid nitrogen and homogenized with phosphate buffered saline (pH 7.4) containing 81 mM Na_2_HPO_4_ and 19 mM NaH_2_PO_4_. The contents of UMP, UDP, UTP, and CTP were examined by corresponding ELISA kits (Mlbio, Shanghai, China) using a micro-ELISA reader RT-6100 (Rayto, Shenzhen, China) equipped with a 450 nm filter.

### 4.6. Measurement of MDA and H_2_O_2_ Contents

The youngest fully expanded leaves were collected from four-week-old rice lines grown in chambers with normal conditions or stress treatment conditions. For measurement of MDA contents, samples were ground in liquid nitrogen using a mortar and pestle into which 5 mL ice-cold 10% (*w*/*v*) trichloroacetic acid had been added. The concentration was measured following the method of Dionisio-Sese and Tobita [54]. For measurement of H_2_O_2_ contents, samples were homogenized in 4 mL of 10 mM 3-amino-1, 2, 4-triazole, and the samples were centrifuged for 25 min at 6000 *g*. The supernatant solution was used to determine the concentration following the method of Brennan and Frenkel [55].

### 4.7. Determination of Antioxidant Enzyme Activities

The youngest fully expanded leaves were collected from four-week-old rice lines grown in chambers with normal conditions or stress treatment conditions. Samples were homogenized in a 5 mL extraction buffer (100 mM sodium phosphate buffer, pH 7.0). The homogenates were centrifuged for 15 min at 10,000× *g* at 4 °C, and then the supernatant solution was collected. The SOD activity was assayed based on inhibition of photoreduction of nitroblue tetrazolium as described by Giannopolitis and Ries [56]. The POD activity was assayed based on the conversion of guaiacol to tetraguaiacol, which was monitored at 470 nm as described by Maehly and Chance [57].

## 5. Conclusions

Our study revealed that *UMPK*, encoding the UMP kinase, is involved in the regulation of PEP-dependent gene transcription and is essential for chloroplast development. Moreover, mutation of *UMPK* leads to over-accumulation of reactive oxygen species and consequently increases sensitivity to cold stress. These results enhance our understanding of the roles of the UMP kinase gene in chloroplast development and abiotic stress response in rice.

## Supplementary Materials

Supplementary materials can be found at https://www.mdpi.com/1422-0067/20/9/2107/s1.

## Abbreviations

Chl	Chlorophyll
Car	Carotenoid
PSI	Photosystem I
PSII	Photosystem II
*∆I/Io*	Content of active P700
*Φ_PSII_*	Actual quantum efficiency
CP	Chloroplast
G	Grana
ROS	Reactive oxygen species
MDA	Malondialdehyde
H_2_O_2_	Hydrogen peroxide
SOD	Superoxide dismutase
POD	Peroxidase
PEP	Plastid-encoded RNA polymerase
NEP	Nucleus-encoded RNA polymerase
