# Supplementary material for: UMP Kinase Regulates Chloroplast Development and Cold Response in Rice
Source: Int J Mol Sci. 2019 Apr 29;20(9):2107. doi: 10.3390/ijms20092107 (PMC6539431; doi:10.3390/ijms20092107)
Supplement: Supplementary file 1 [file ijms-20-02107-s001.pdf]

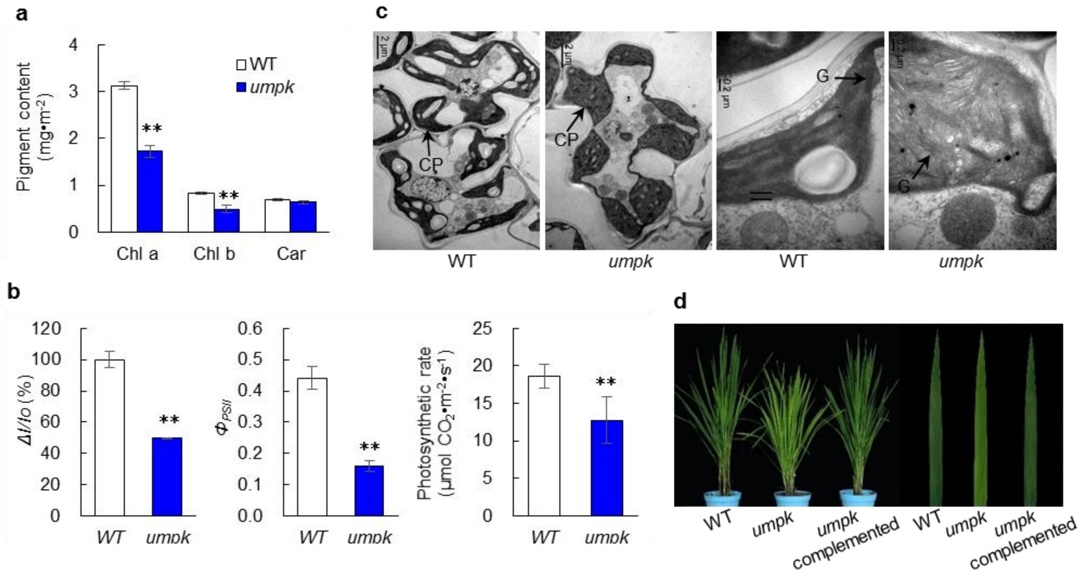

Figure S1. Phenotypic characterization of WT, the *umpk* mutant, and the transgenic plants. (**a**) Concentrations of chlorophyll (Chl) and carotenoid (Car) in WT and the mutant. (**b**)The light-induced P700 absorbance changes at 820 nm (*ΔI/Io*), the actual quantum efficiency (*ΦPSII*), and photosynthetic rate of WT and the mutant. (**c**) Transmission electron microscope analysis of WT and the mutant. CP, chloroplast; G, grana. (**d**) (**b**) Phenotypic complementation of the *umpk* mutant by introducing of the *UMPK* gene of WT. Data represent means ± sd (*n* = 5). Significant difference according to the Student's t test at * $P < 0.05$ and ** $P < 0.01$.

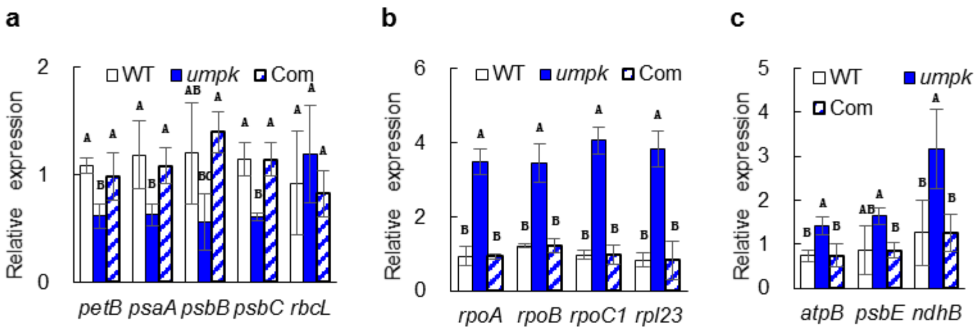

Figure S2. Expression of plastid genes in the complementation lines (Com) and the *umpk* mutant. (**a**) Plastid-encoded RNA polymerase (PEP)-dependent genes. (**b**) Nucleus-encoded RNA polymerase (NEP)-dependent genes. (**c**) Both PEP- and NEP-dependent genes. The *Actin1* was used as an internal control. Data represent means ± sd (n = 3). Bar with different letters are significantly different at $P < 0.01$ based on Duncan's multiple range tests.

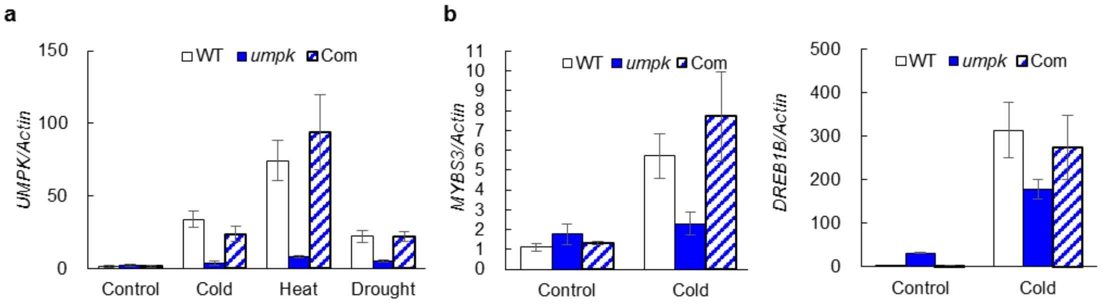

Figure S3. Response of *UMPK* to abiotic stresses in WT, the mutant, and the complementation lines. (**a**) Expression levels of *UMPK* under various stress treatments. (**b**) Expression levels of two cold inducible genes under control and cold conditions. $n = 3$, *Actin1* was used as the internal control. Data represent means ± sd.

Table S1 Primers used in this study.

Name	Forward primer (5'-3')	Reverse primer (5'-3')
**Fine-mapping**		
D1	GCGTGCTTTTGATCCGTTG	TGCTCCCGGACGTACACTC
D2	TGGGTACTGTAGCTCATTGTC	CCTTCCGTAAACTTGATCGTC
D3	ATGCAACTTTGGTGATTTGGAA	AAAATGATTAGCCACCCACAT
D7	TTACAATTATTAGCCACCACTTC	ATTACGAGCATTACTTGTGTA
Q1	TCACAGGTAGACTGGTAGTGTTT	GTATGTTTCGTGCGGTAACTT
Q3	ATTCCTCAATTACTTCTATCAGC	CATCATCAGTGAGTTGGGTA
Q4	AGGAAACTAAATAAAGCGACAC	TGCACGGTTGTTATTGGCAC
Q5	ATGAAATGCTCACTTACGTCT	TCCCAATTGCAAAATTACACATT
**Vector construction**		
Complementation	CGGTACCCGGGGATCCCGGACTCCCA GATACGCAAC	CCAAGCTTGCATGCCTGCAGAGAACAATC CGAAGCACGAGA
**Real-time PCR analysis**		
UMPK	GATTACATTGGCATGTTGGCTAC	CTGCATTGATTTCTGCACAACG
OsNDPK2	CTTTTTCAGTGCCCCAAGGA	TCGATCAGACCGGGAAAGAA
psaA	GCGAGCAAATAAAACACCTTTC	GTACCAGCTTAACGTGGGGAG
psbB	TAGTTTCTGGTTGGGCTGGCTC	CTCCAACCACCCCACGAATTG
psbC	GTTCCCCAACGGGAGAGGTTAT	GAGCCTAAAGGAGCATGGGTCAT
petB	TTCAGACCTCGCAACCAGACTG	AACAAAAGGCAAGGGTTCTTCGA
rbcL	CTTGGCAGCATTCCGAGTAA	ACAACGGGCTCGATGTGATA
rpoA	CGTGGGTTCTGTCAAGTTAGCTATAG	GGTCCTAGATACATAACTGCCCAAGA
rpoB	CGAGATATCCATCCGAGTCACTATG	GCTAAAGATCCAGTAAGTCCAACGT
rpoC1	AATCTCCTATCTCCCGCTATTGG	CAATCGTTAATACATAAAGTCCGATAAGC
rpl23	GGGTCGAACTCTTCTTTGGTGTT	GGGCCCATTCTTCTACCCTTT
atpB	TCGCAATTCTTGGGTTGGATGA	CAACATACTTTCCCGGAGAACCG
ndhB	GCGCTTATATCCATCACTGTAGGAC	CACTCCTTCGTAGACGTCAGGA
psbE	TGTCTGGAAGCACGGGAGAACGT	GTTTGGCCGAGGACTTCCAAACAC
MYBS3	CCTTTCTGGCAAAATCAGAAAGA	ATGAACTGGAACAGGCTTGACA
DREB1B	AGCTCGCCGGCTCCGACA	GGGAGAAATCTGGCACATTCC
